# Locating the anterior interosseous nerve in relation to the surgically relevant landmarks of the forearm: A cadaveric study

**DOI:** 10.1016/j.amsu.2021.102930

**Published:** 2021-10-12

**Authors:** Vrinda H. Ankolekar, Mamatha Hosapatna, Anne Dsouza

**Affiliations:** Department of Anatomy, Kasturba Medical College, Manipal, Manipal Academy of Higher Education, Manipal, India

**Keywords:** Cadaver, Forearm, Landmark, Nerve, Nerve transfers

## Abstract

**Background:**

This study emphasizes locating the anterior interosseous nerve (AIN) related to its level of origin, number, and lengths of its muscular branches and relation to surgically important landmarks such as the bi-epicondylar line, pronator teres, and Gantzer muscles.

**Methods:**

The study was undertaken at a private Medical school in south India during 2019–20. The level of origin of AIN was measured from the bi-epicondylar line and its length was measured up to the upper border of the pronator quadratus using 44 cadaveric upper limbs. The number of branches given to flexor digitorum profundus (FDP) and flexor pollicis longus (FPL) was quantified and their lengths were measured.

**Results:**

The nerve originated at a mean distance of 41.56 mm from the bi-epicondylar line. In 12 upper limbs, FDP received two branches and in two limbs, it received three branches. In 13 upper limbs, FPL received two branches from AIN. It was observed that the muscular branches for FDP were shorter than those for FPL. Gantzer muscle was observed in 18 (40%) specimens and was found superficial to the nerve.

**Conclusion:**

The muscular branches of AIN had a variable pattern of innervation. Multiple muscular branches to the FPL and FDP were observed in the upper 2/3rds of the forearm. These branches to FPL and FDP would aid as a source of nerve grafting and nerve transfer in the cases of upper extremity nerve palsies.

## Highlights

• AIN concerning the surgically relevant landmarks such as bi-epicondylar line, pronator teres (PT) were explored.
• The AIN originated at a mean distance of 41.56 mm from the bi-epicondylar line.
• It was observed that the muscular branches for FDP were shorter than those for FPL.
• The muscular branches of AIN had a variable pattern of innervation.
• The branches to FPL and FDP would aid as a source of nerve grafting and nerve transfer surgeries.

## 1 Introduction

The Anterior Interosseus Nerve (AIN) arises distal to the bi-epicondylar line and innervates the deep flexors of the forearm [1].

AIN syndrome accounts for not more than one percent of all upper extremity nerve palsies. These nerve palsies arise due to either nerve compression or inflammation [2]. Lesions of this nerve commonly occur as a complication following intravenous infusion, leading to the oedema of the forearm. Entrapment of AIN underneath the pronator teres is also reported in the literature [3]. The AIN is also often used for nerve transfer surgeries for the treatment of forearm nerve injuries [4,5].

Literature reveals a variable incidence of the presence of Gantzer muscle, which is considered as the accessory head of ‘flexor pollicis longus’ (FPL) or flexor digitorum profundus (FDP) [6,7]. Gantzer muscle is considered as one of the vulnerable compression points for AIN [8,9].

The AIN nerve is often exposed during surgical decompression and nerve transfer surgeries and hence its course related to the surgical landmarks has remarkable significance. Therefore, the present study was carried out to explore the origin and course of AIN concerning the surgically relevant landmarks such as bi-epicondylar line, pronator teres (PT), and Gantzer muscles. The study also emphasizes the quantification of branches to the FPL and FDP.

## 2 Materials and methods

The study was carried out in a cross-sectional manner using twenty-two formalin-fixed adult cadavers (i.e., 44 upper limbs) over one year during 2019-20 with approval from the Institutional Ethics Committee (IEC 26/2019). The average age of the cadavers ranged between 40 and 65 years. There were 18 male and four female cadavers. Upon the dissection of the forearm, the superficial, deep flexor muscles and the median nerve were identified. We identified the point of origin of the AIN and its branches to the FDP and FPL.

**Fig. 1:**
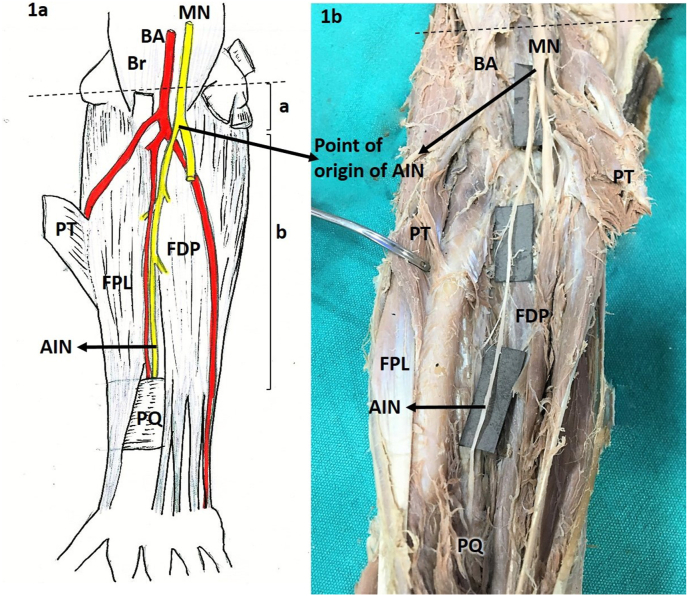
a: Drawing showing the origin and course of the AIN in the forearm Fig. 1b: Cadaveric specimen of the right upper limb showing the AIN and its coursea. Distance measured from the bi-epicondylar line (dotted line) up to the origin of AIN b. Length of the AIN from its origin up to the upper border of PQ AIN- Anterior interosseous nerve, PT- Pronator teres, MN- Median nerve, FDP- Flexor digitorum profundus, FPL- Flexor pollicis longus, Br- Brachialis, BA- Brachial Artery, PQ- Pronator quadratus.

We measured the length of the AIN from the point of its origin from the median nerve up to the bi-epicondylar line, and also from its origin to the upper border of the pronator quadratus (PQ) muscle (Fig. 1). Its course, either deep or between the two heads of PT was also observed. We noted the direction of origin of the AIN, whether anterior or posterior. We then quantified the number of branches given by the AIN to FDP and FPL. The lengths of those branches were measured from their origin to the point of penetration into the muscle using digital calipers. The length of the forearm in each specimen was measured from the bi-epicondylar line to the ulnar styloid process. The relation of AIN to the Gantzer muscle when present was also observed. The data were analyzed using Microsoft Excel (© Microsoft 2020) for the mean and standard deviation. We then correlated the length of the forearm with the distance of the origin of AIN using Pearson's correlation test. A correlation coefficient >0.5 was considered to be significant.

## 3 Results

### 3.1 Origin and course of AIN

AIN was present in all the cadaveric upper limbs observed. The nerve was best identified, after retracting the PT muscle, which served as an important landmark in the upper forearm. In the middle of the forearm, the AIN was found to be deep to the Gantzer muscle, and later it traversed laterally to it. It then traversed underneath the flexor digitorum superficialis (FDS). In the lower third, the nerve was best identified between the two deep flexor muscles (FDP and FPL) in close contact with the interosseous membrane and passed deep to the PQ at its upper border.

Out of 44 upper limbs observed, AIN originated deep into the two heads of PT in 39 specimens, and in between the two heads of PT in two specimens. In three upper limbs, the origin of AIN was distal to the PT muscle. In all the upper limbs observed, the AIN originated from the posterior aspect of the median nerve.

**Fig. 2:**
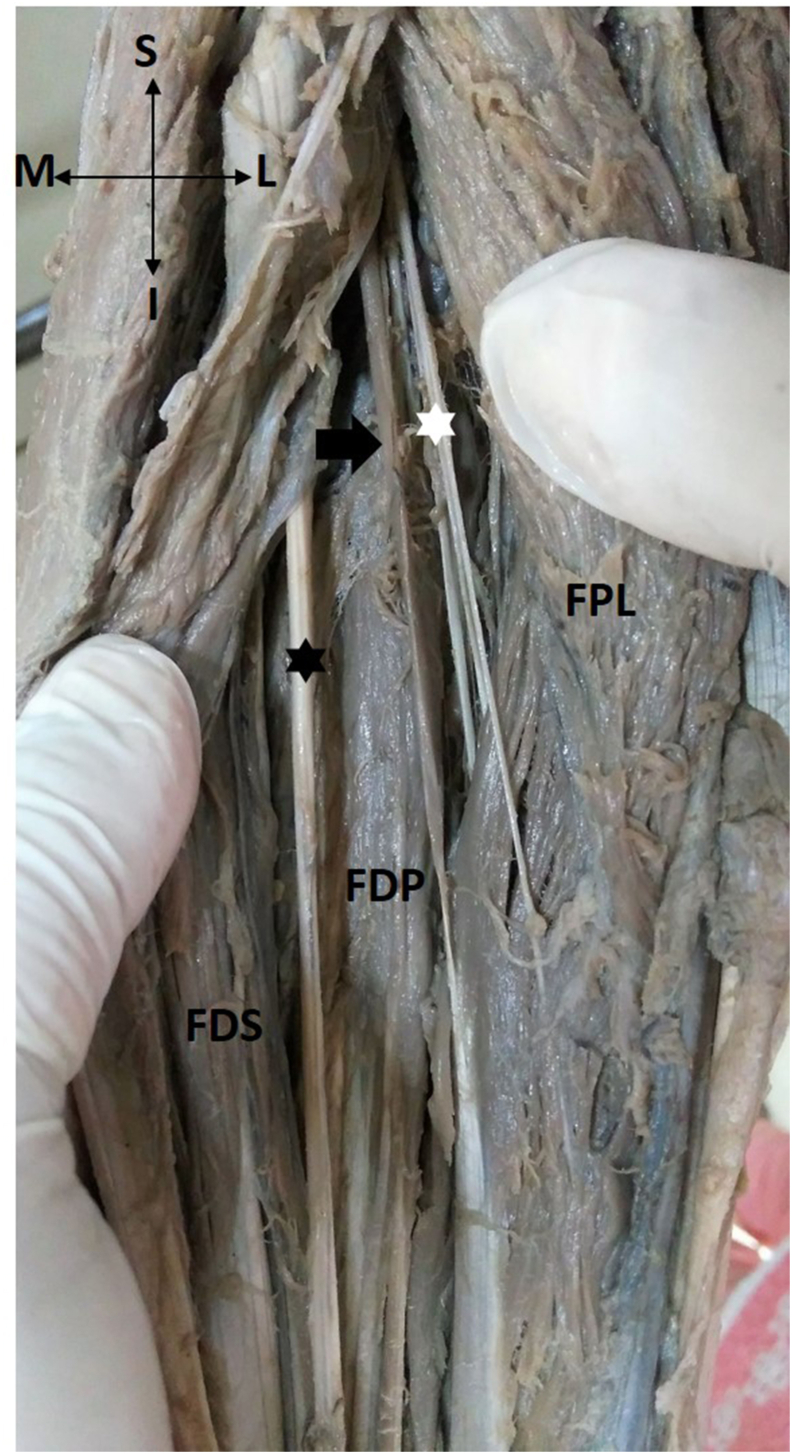
Photograph of the forearm, after retracting flexor pollicis longus (FPL) and flexor digitorum superficialis (FDS) showing the relation of AIN (white asterisk) to the Gantzer muscle (black arrow). MN- Median nerve (black asterisk), FDP- flexor digitorum profundus.

Gantzer muscle was observed in 18 upper limbs (40%) (Fig. 2). In all 18 specimens, the Gantzer muscle was located superficial to the AIN, crossing it from medial to lateral in the middle 1/3rd of the forearm.

The branches of the AIN innervated FDP and FPL in the upper 2/3rds of the forearm. It was observed that the muscular branches for FDP were shorter than those for FPL.

### 3.2 Biometry of AIN

**Fig. 3:**
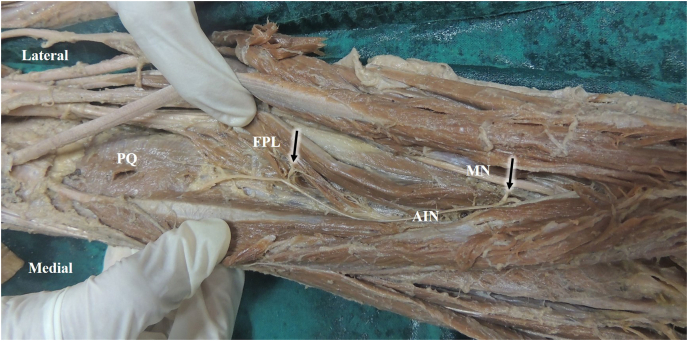
Innervation of FPL through two muscular branches of AIN (Black arrows) MN- Median nerve, AIN-Anterior interosseous nerve, PQ-Pronator quadratus, FPL- Flexor pollicis longus.

The lengths of the upper limbs ranged from 23 to 30 cm, with a mean of 27.31 cm. The AIN originated at a mean distance of 4.16 cm from the bi-epicondylar line (range 2.4 cm–6 cm). The mean length of the AIN was found to be 14.26 cm (SD 2.09 cm), and the length ranged from 11.10 cm to 19.80 cm. In 30 upper limbs, FDP received a single muscular branch from AIN with an average length of 2.88 cm (range 1.80 cm–4.00 cm). In 12 upper limbs, FDP received two branches from the AIN with mean lengths of 2.81 cm and 3.48 cm. In two upper limbs, FDP received three branches from the AIN at the mean distances of 0.9 cm, 1.45 cm, and 2.30 cm, respectively. In 31 upper limbs, the FPL received a single muscular branch from AIN with an average length of 4.26 cm (range 1.20 cm–8.00 cm). In 13 upper limbs, FPL received two branches from the AIN, with mean lengths of 3.16 cm and 3.34 cm (Fig. 3). There was a weak negative correlation between the length of the forearm and the distance of the origin of the AIN (correlation coefficient −0.1). Table 1 shows the detailed description of branches to the FDP and the FPL muscles.

**Table 1:** Detailed description of the upper limbs observed for the number of muscular branches to the FDP and the FPL.

Branches to the Flexor Digitorum Profundus (FDP)						
	One branch (N = 30)	Two branches (N = 12)		Three branches (N = 2)		
Mean distances (cm)	2.88	2.81	3.48	0.9	1.45	2.3
**Branches to the Flexor Pollicis Longus (FPL)**						
	One branch (N = 31)	Two branches (N = 13)				
Mean distances (cm)	4.26	3.16	3.44			

## 4 Discussion

The course of the AIN with the important surgical landmarks has been elaborated in the current study. The biometry of the nerve and its muscular branches are the highlights of this study.

There are several studies in the literature using the distal branches of AIN for nerve transfer surgeries in cases of upper extremity nerve palsies [10,11]. The Gantzer muscle is one of the sites of compression of the AIN [12].

The literature reveals several anatomical studies describing the anatomy of the AIN [5,[10], [11], [12], [13]]. Tubbs et al. in their study on ten upper limb specimens noted that the AIN originated at a distance of 5.4 cm from the medial epicondyle of the humerus [5]. Canovas et al. observed a more proximal origin, of 4.3 cm from the bi-epicondylar line [13]. Vincelet et al. observed the origin of the AIN at an average distance of 4.5 cm from the bi-epicondylar line [14]. In the current study, the origin of AIN was noted at a mean distance of 4.16 cm from the bi-epicondylar line which was slightly lesser than the values mentioned in the existing literature.

Yamada et al. undertook cadaveric dissections to determine any anatomical factors predisposing to AIN injuries such as venepuncture procedures [15]. Mathieu et al., in a study on 12 cadavers, observed that the commonest location of the AIN was beneath the PT (nine specimens). In two specimens, it was located proximal to the PT, and in one specimen, it was distal to the PT. They also observed that the relative positions of AIN branches were variable [7]. In the current study, AIN originated deep to the two heads of PT in 39 specimens, and in between the two heads of PT in two specimens. However, in the present study, in three upper limbs, the origin of AIN was distal to the PT muscle.

Vincelet et al. observed that the origin of the AIN was from the posterior aspect of the median nerve at a distance of 4.5 ± 1.36 cm [15]. The literature reveals a variable description of the origin of AIN, as it originates either from the posterior or the lateral side of the median nerve [11,13,16]. In contrast to these, few studies show its origin from the medial side of the median nerve [17]. However, in the present study, AIN originated from the posterior aspect of the median nerve in all the specimens observed.

Canovas et al. reported less variability in the branches innervating FDP. They also measured the mean distances of 5.9 cm from the bi-epicondylar line [13]. However, the present study disclosed a variable pattern of innervation. In 12 upper limbs, FDP received two branches, and in two limbs, it received three branches. In 13 upper limbs, FPL received two branches from AIN. It was also observed that the muscular branches for FDP were shorter than those for FPL.

A study was done by Caetano et al. on 80 upper limbs, revealed the presence of Gantzer muscle in 54 limbs (68%) [6]. Yang et al. in their study on 73 upper limbs, reported that the Gantzer muscle was present in 35 limbs (47.95%) [7]. In the current study, the incidence of Gantzer muscle was found to be in 18 upper limbs (40%) which is close to the incidence found by Yang et al.

The mean length of the AIN available for transfer would aid a surgeon plan the nerve transfer in case of ulnar nerve injuries [18]. The AIN branches can be easily used for nerve transfer surgeries of the median and ulnar nerves [18]. Therefore, an understanding of the morphometry of the AIN and its branches would help surgeons during nerve transfer surgeries.

Even though the literature reveals several studies on the anatomy of the AIN related to the PT muscle, in the present study, the lengths of particular muscular branches to FPL and FDP were measured additionally. However, due to the less number of female cadavers, the biometry of the AIN was not compared between males and females using inferential statistics and we did not compare the values between the right and left sides which could be considered as limitations of our work. Formalin fixation tend to influence the tissue morphometry and hence the findings of the present study can be substantiated using fresh-frozen cadavers which is a future scope for research.

## 5 Conclusion

Multiple muscular branches to the FPL and FDP were observed in the upper 2/3rds of the forearm. The course of AIN, in relation to the surgical landmarks, would provide a guide for the surgeon to identify the compression site. The landmarks could also serve as a guide during decompression surgeries. The multiple muscular branches to the FPL and FDP would aid as a source of nerve grafting and nerve transfer in the cases of upper extremity nerve palsies. The knowledge of the pattern of innervation of FPL and FDP would also be essential for the choice of the nerve, which will be used for transfer.

## Conference presentation

Dr Mamatha Hosapatna has presented a part of this research project at during International Virtual Anatomy Conference organized by the Anatomical Society, King George's Medical University, Lucknow, UP from 20th to February 22, 2021.

## Annals of medicine and surgery

The following information is required for submission. Please note that failure to respond to these questions/statements will mean your submission will be returned. If you have nothing to declare in any of these categories then this should be stated.

The authors state that there is no conflict of interest to declare.

## Ethical approval

Research studies involving patients require ethical approval. Please state whether approval has been given, name the relevant ethics committee and the state the reference number for their judgement.

Approval from the Institutional Ethics Committee has been obtained before the commencement of the study (IEC 26/2019).

## Please state any sources of funding for your research

All sources of funding should be declared as an acknowledgement at the end of the text. Authors should declare the role of study sponsors, if any, in the collection, analysis and interpretation of data; in the writing of the manuscript; and in the decision to submit the manuscript for publication. If the study sponsors had no such involvement, the authors should so state.

This study did not receive any funding from external agencies.

## Author contribution

Please specify the contribution of each author to the paper, e.g. study concept or design, data collection, data analysis or interpretation, writing the paper, others, who have contributed in other ways should be listed as contributors. Vrinda HA and Mamatha H conceptualized and designed the study, conducted research, provided research materials, and collected and organized data. Anne D Souza analyzed and interpreted data. Mamatha H & Anne wrote initial draft and Vrinda HA scrutinized it further provided logistic support. All authors have critically reviewed and approved the final draft and are responsible for the content and similarity index of the manuscript.

## Consent

Studies on patients or volunteers require ethics committee approval and fully informed written consent which should be documented in the paper.

Authors must obtain written and signed consent to publish a case report from the patient (or, where applicable, the patient's guardian or next of kin) prior to submission. We ask Authors to confirm as part of the submission process that such consent has been obtained, and the manuscript must include a statement to this effect in a consent section at the end of the manuscript, as follows: "Written informed consent was obtained from the patient for publication of this case report and accompanying images. A copy of the written consent is available for review by the Editor-in-Chief of this journal on request”.

Patients have a right to privacy. Patients’ and volunteers' names, initials, or hospital numbers should not be used. Images of patients or volunteers should not be used unless the information is essential for scientific purposes and explicit permission has been given as part of the consent. If such consent is made subject to any conditions, the Editor in Chief must be made aware of all such conditions.

Even where consent has been given, identifying details should be omitted if they are not essential. If identifying characteristics are altered to protect anonymity, such as in genetic pedigrees, authors should provide assurance that alterations do not distort scientific meaning and editors should so note.

Not applicable.

## Registration of research studies

In accordance with the Declaration of Helsinki 2013, all research involving human participants has to be registered in a publicly accessible database. Please enter the name of the registry and the unique identifying number (UIN) of your study.

You can register any type of research at http://www.researchregistry.com to obtain your UIN if you have not already registered. This is mandatory for human studies only. Trials and certain observational research can also be registered elsewhere such as: ClinicalTrials.gov or ISRCTN or numerous other registries.

## Guarantor

The Guarantor is the one or more people who accept full responsibility for the work and/or the conduct of the study, had access to the data, and controlled the decision to publish.

## Declaration of competing interest

The authors do not have any conflict of interest to declare.

## Abbreviations

AIN: anterior interosseous nerve
FDP: flexor digitorum profundus
FPL: flexor pollicis longus
IEC: institutional Ethics Committee
PQ: pronator quadratus
PT: pronator teres
